# Neoadjuvant Chemotherapy for Early Breast Cancer: A Study on Response Rate and Toxicity

**DOI:** 10.3390/jcm14207362

**Published:** 2025-10-17

**Authors:** Matt Galloway, Paula Barlow, Jody Jordan, Edward Lo

**Affiliations:** Department of Medical Oncology, Fallen Soldiers’ Memorial Hospital, Hawke’s Bay, Hastings 4120, New Zealand; paula.barlow@hbdhb.govt.nz (P.B.); jody.jordan@hbdhb.govt.nz (J.J.); ed.lo@hbdhb.govt.nz (E.L.)

**Keywords:** neoadjuvant chemotherapy, early breast cancer, chemotherapy toxicity, chemotherapy response rate

## Abstract

**Background**: Neoadjuvant chemotherapy (NACT) is widely used in patients with high-risk HER2-amplified (HER2+) or triple negative early breast cancer (TNBC). Advantages of NACT include allowing less extensive surgery, assessing response to treatment and guiding adjuvant therapy. NACT-related toxicities are common and can result in treatment alterations and hospitalisation, which may adversely impact outcomes. **Aim**: To assess NACT treatment in Hawke’s Bay (HB), New Zealand, by evaluating pathologic complete response (pCR) rates and toxicities of different regimens. **Method**: Data were retrospectively obtained from medical records of NACT patients. pCR rates were compared to results from the previous literature. Toxicity was assessed by recording severe (grade 3 or above) toxicities, treatment-limiting toxicities (those leading to dose reductions, dose delays or early cessation) and hospitalisations for different NACT regimens. **Results**: A total of 71 NACT patients were included. pCR rates for HER2+ disease and TNBC were 19/45 (42%) and 8/24 (33%), respectively. The most common severe toxicities were diarrhoea, anaemia and febrile neutropaenia (all 16%) in FEC-D (5-fluorouracil/epirubicin/cyclophosphamide + docetaxel +/− carboplatin +/− immunotherapy) patients, neutropaenia (50%) in FEC-DH (FEC-D + trastuzumab +/− pertuzumab) patients and diarrhoea (38%) in TCH (docetaxel/carboplatin/trastuzumab +/− pertuzumab) patients. Comparing treatment-limiting toxicity in FEC-DH vs. TCH, 9/16 (56%) vs. 13/21 (62%) had dose reduction, 2/16 (13%) vs. 8/21 (38%) had dose delay, 1/16 (6%) vs. 5/21(24%) had early cessation and 6/16 (38%) vs. 13/21 (62%) were hospitalised, respectively. **Conclusions**: NACT was associated with high rates of severe and treatment-limiting toxicity. Despite this, pCR rates were consistent with the previous literature. With the caveat of small patient numbers, FEC-DH-based therapy was associated with fewer dosing delays, early cessations and hospitalisations compared with TCH-based therapy.

## 1. Introduction

Treatment of breast cancer prior to definitive surgery, known as neoadjuvant therapy, is used increasingly as an approach for managing early breast cancer [1]. There is well-recognised evidence that supports the use of neoadjuvant chemotherapy (NACT) in clinically high-risk human epidermal growth factor receptor 2 positive (HER2+) breast cancer and triple negative breast cancer (TNBC) [2]. Research demonstrates that the addition of HER2+ targeted therapies or immunotherapy can also improve outcomes in these patients [3,4].

While NACT does not improve overall survival or risk of distant disease recurrence when compared with adjuvant therapy [5], it can provide important benefits. These include downstaging disease, making breast-conserving surgery (BCS) possible, and allowing treatment response to be assessed, which can in turn aid prognostication and guide adjuvant therapy [2]. Compared with adjuvant therapy, NACT has been associated with increased rates of local recurrence, possibly due to increased use of BCS and some good responders to NACT not undergoing definitive surgery [6], however this does not seem to increase risk of distant disease recurrence or mortality [5].

The consideration of toxicity when selecting a NACT regimen is of significance to both the patient and oncologist. Patients who experience significant toxicity from NACT can have their surgery delayed [7], which has been associated with poorer outcomes [8]. In addition, toxicity can also lead to a reduction in dose intensity via dose delays or reductions, which has been shown to negatively impact overall survival [9,10].

Pathologic complete response (pCR) is considered a useful objective outcome measure in neoadjuvant research for breast cancer as it has been shown to correlate with improved overall survival [11,12]. The definition of pCR used in past research has varied, however it has been demonstrated that achieving pCR in both the breast and axillary nodes is associated with improved event-free survival (EFS) and overall survival (OS) compared with pCR in breast only [13]. Furthermore, patients with residual ductal carcinoma in situ (ypT0/is ypN0) have equivalent EFS and OS compared to patients with no tumour cells remaining in the breast (ypT0 ypN0) [14], and therefore ‘ypT0/is ypN0’ has been considered a preferred pCR definition [15]. Achieving pCR following NACT is not only of prognostic significance but also clinical significance, as patients with residual disease are recommended to receive additional adjuvant systemic treatment with either trastuzumab emtansine in HER2+ disease [16] or capecitabine in TNBC [17] to reduce risk of recurrence.

Current guidelines recommend an anthracycline and taxane-based combination for NACT in TNBC [18]. This has stemmed from past adjuvant research, which shows improved invasive disease-free survival in anthracycline-based regimens compared to the non-anthracycline-based alternative [19]. Subsequent evidence in the neoadjuvant setting demonstrates improved pCR rates with the addition of a taxane therapy [1]. pCR rates can be further improved by the addition of carboplatin, with a suggestion that this may also improve EFS [12,20]. Recent evidence has emerged highlighting that the inclusion of pembrolizumab (an immunotherapy) can improve pCR rates, EFS and OS in TNBC patients [4,21].

For HER2+ breast cancer, there are various NACT regimens which are considered appropriate, with a lack of clear evidence demonstrating superiority in EFS and OS between regimens [22]. All treatments should include the targeted HER2 hormonal agent trastuzumab, as this has produced better response rates without significant increase in toxicity [23]. The addition of a second HER2 agent (pertuzumab) has been found to improve pCR rates, although this has not yet translated into significant improvement in survival [1].

As in TNBC, historical treatment of early HER2+ breast cancer was commonly based on an anthracycline/taxane combination [24]. However, studies that compared anthracycline-containing regimens with non-anthracycline-containing regimens found equivalent pCR, EFS and OS rates between groups and found increased toxicity in the anthracycline-containing group, more specifically in rates of febrile neutropaenia and decline in left ventricular ejection fraction (LVEF) [25]. Both anthracyclines and trastuzumab are known to have cardiotoxic effects characterised by a reduction in LVEF [26,27], which has led to concern regarding their combined use in HER2+ patients. There is also concern of slight increased risk in haematological malignancy in the anthracycline-containing regimens [28,29].

Influenced by the above research, the preferred HER2+ NACT regimen in Hawke’s Bay (HB), New Zealand, has shifted from the anthracycline-containing regimen FEC-DH (5-fluorouracil, epirubicin and cyclophosphamide followed by docetaxel alongside trastuzumab) to the non-anthracycline-containing regimen TCH (docetaxel, carboplatin and trastuzumab) in recent years. For TNBC, the preferred treatment regimen is FEC-DC (5-fluorouracil, epirubicin and cyclophosphamide followed by docetaxel and carboplatin). The aim of this audit was to assess NACT treatment in HB by evaluating pCR rates and toxicities of the different regimens.

## 2. Method

### 2.1. Patient Recruitment

After receiving ethical approval from the local research committee, a search was conducted within the MidCentral Regional Cancer Treatment Service (RCTS) electronic database to identify patients who underwent NACT for early breast cancer. Patients who were treated in the HB district were identified for inclusion.

### 2.2. Data Collection

The electronic oncology clinic letters and chemotherapy prescribing system were used to extract data on patient demographics, molecular subtype, chemotherapy regimens, pCR, treatment limitations (dose delay, reduction and early cessation) and toxicities. Data on molecular subtype and pCR were recorded from histology reports. Treatment limitations were assessed by reading oncology clinic letters and noting incidences of dose delay, reduction and early cessation. Toxicities that were documented in clinic letters as the cause of treatment limitations were recorded. ‘Severe’ toxicity data was collected and included grade 3 or above toxicity, as per the Common Terminology Criteria for Adverse Events (CTCAEs) system [30], and toxicity that led to hospitalisation. In cases where toxicity documentation was insufficient to grade severity on the CTCAE system (e.g., number of stools not documented for diarrhoea) then toxicity labelled ‘severe’ by the treating oncologist in clinic letters was also included.

### 2.3. Process Clarifications and Definitions

For this audit, pCR was defined as ypT0/is ypN0 based on recommendations from relevant studies [15], and therefore ductal carcinoma in situ on the post-operative histology was regarded as pCR. For toxicity documentation, when symptoms were attributed to a named diagnosis (e.g., fever, cough, dyspnoea caused by lung infection) then the diagnosis was recorded rather than each individual symptom. In some instances, symptoms were attributed to other medications (such as aches/bone pain due to pegylated Filgrastim) and these were not recorded as chemotherapy toxicity. The three most common treatment groups (FEC-D, FEC-DH and TCH) used identical supportive medication protocols consisting of multiple antiemetics and pegylated Filgrastim.

‘Dose reductions’ included reduced doses of chemotherapy drugs, stopping drug components for the next cycle (e.g., stopping carboplatin but continuing docetaxel in FEC-DC) and not escalating treatment to maximum dosing due to toxicity (e.g., in FEC-D and FEC-DH, where standard practice is to begin docetaxel at 75 mg/m^2^ and then increase to 100 mg/m^2^ for cycles 5 and 6 if well tolerated). ‘Treatment delay’ was a delay in treatment for at least one week and ‘early cessation’ was recorded for anyone who did not complete the total number of planned treatment cycles.

## 3. Results

**Figure 1:**
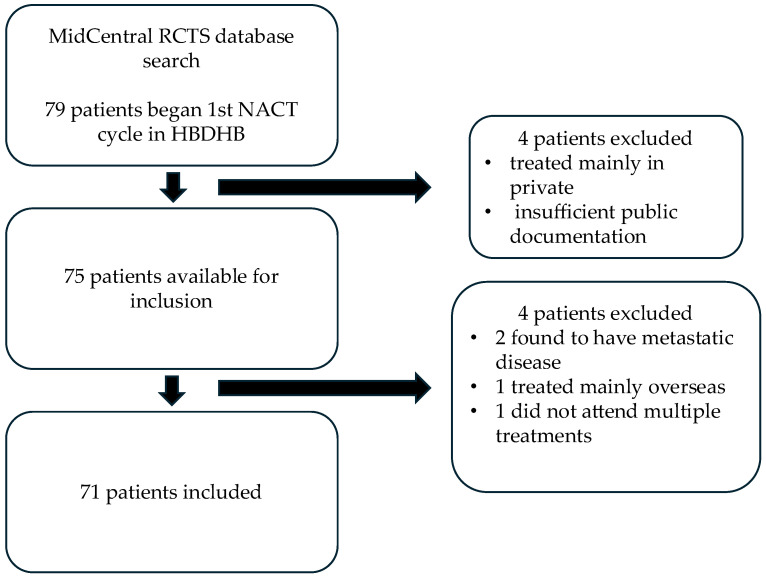
Inclusion criteria.

Between 1 January 2019 and 31 January 2025, a total of 79 patients commenced NACT treatment in HB (Figure 1). Four patients were treated mainly privately and due to insufficient public documentation available they could not be included. A further four patients were subsequently excluded from final analysis: two were found to have metastatic disease on delayed staging scans, one received the majority of their treatment overseas and the available documentation was inadequate and one did not attend NACT treatments and developed metastatic disease. Ten of the included patients also received additional neoadjuvant HER2+ therapy or immunotherapy privately.

All 71 patients included were female and aged between 29 and 80 years, with a median age of 53 years. The breakdown of patients per molecular receptor type was 24 (34%) with TNBC, 44 (62%) with HER2+ disease and 3 (4%) with hormone receptor positive/HER2 negative (HR+/HER2−) disease. One HER2+ patient received two different chemotherapy regimens due to severe toxicity to the first regimen, and therefore the pCR and toxicity data for both regimens are included.

### 3.1. pCR Rates

Table 1 shows the pCR rates per molecular subtype, with the highest rates in the HER2+/HR− group (62%) followed by the HER2+/HR+ group (34%) and the TNBC group (33%). Table 2 is a record of the various NACT regimens used and the pCR rates for each regimen. In this study, the regimens have been grouped into broader categories based on a common treatment base. These are ‘non FEC-D’-based (doxorubicin and cyclophosphamide followed by paclitaxel, or ‘AC + P’; and carboplatin, paclitaxel and pembrolizumab followed by doxorubicin and cyclophosphamide, or ‘C/P/Pem + AC’), ‘FEC-D’-based (FEC-D, FEC-DC and FEC-DC +/− pembrolizumab or atezolizumab), ‘FEC-DH’-based (FEC-DH +/− pertuzumab), ‘P/H’-based (paclitaxel and trastuzumab, or ‘P/H’, +/− pertuzumab; and doxorubicin and cyclophosphamide followed by paclitaxel and trastuzumab, or ‘AC + P/H’) and ‘TCH’-based (TCH +/− pertuzumab) treatment. Twenty-five patients underwent FEC-D-based treatment, with eight (32%) achieving pCR, sixteen patients underwent FEC-DH-based treatment, with 7 (43.8%) achieving pCR, eight patients underwent P/H-based treatment, with five (62.5%) achieving pCR, and twenty-one patients underwent TCH-based treatment, with seven (33.3%) achieving pCR.

### 3.2. Severe NACT Toxicity

Toxicity analysis was conducted using the three largest treatment base groupings rather than the individual NACT regimens to enable meaningful comparison between groups. Table 3 displays the Eastern Co-operative Oncology Group (ECOG) performance status classification [31] for patients in each of these treatment groups. Table 4 outlines the severe toxicity data for the FEC-D, FEC-DH and TCH treatment base groups. The total number of patients to experience severe toxicity for each group was as follows: 16/25 (64%) for the FEC-D group, 12/16 (75%) for the FEC-DH group and 14/21 (67%) for the TCH group. In the FEC-D group, the most common severe toxicities were diarrhoea, anaemia and febrile neutropaenia, which each occurred in four (16%) patients. In the FEC-DH group, the most common severe toxicities were afebrile neutropaenia, aches and fever, occurring in eight (50%) patients, three (19%) patients and three (19%) patients, respectively. In the TCH group, the most common severe toxicities were diarrhoea, anaemia and mucositis, occurring in eight (38%) patients, four (19%) patients and three (14%) patients, respectively.

### 3.3. Treatment-Limiting NACT Toxicity

Table 5 displays the treatment-limiting toxicities (toxicities causing dose reduction, dose delay or early cessation) for each NACT treatment base. The number of patients who had treatment-limiting toxicity in each group was 12/25 (48%) for the FEC-D group, 13/16 (81%) for the FEC-DH group and 16/21 (76%) for the TCH group. The toxicities that most commonly led to treatment limitations in the FEC-D group were fatigue, diarrhoea and febrile neutropaenia, which occurred in five (20%) patients, four (16%) patients and four (16%) patients, respectively. In the FEC-DH group, the most common treatment-limiting toxicities were aches and diarrhoea, which occurred in four (25%) patients and three (19%) patients respectively. In the TCH group, the most common treatment-limiting toxicities were diarrhoea, fatigue and neuropathy, which occurred in five (24%) patients, four (19%) patients and four (19%) patients respectively. Cardiotoxicity (decline in LVEF) led to treatment limitations in two (13%) FEC-DH-based patients and two (10%) TCH-based patients.

**Figure 2:**
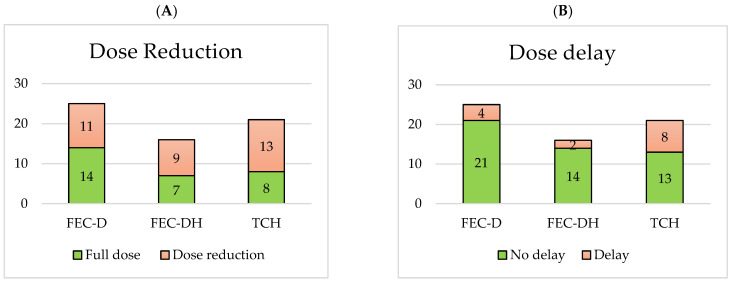

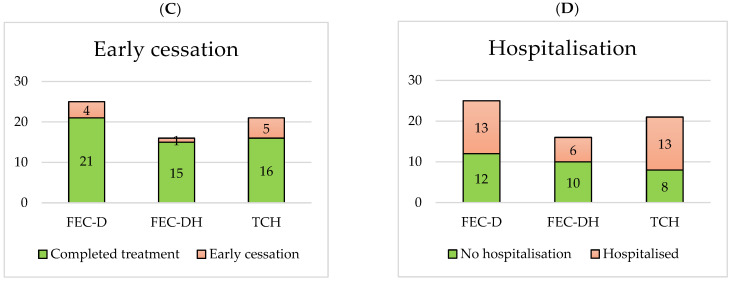
Number of dose reductions (**A**), dose delays (**B**), early cessations (**C**) and hospitalisations (**D**) per NACT treatment base.

The rates of dose reductions, dose delays, early cessation and hospitalisation for each NACT treatment base are displayed in Figure 2. Comparing the HER2+ treatment base groups, 9/16 (56%) FEC-DH patients vs. 13/21 (62%) TCH patients required a dose reduction, 2/16 (13%) FEC-DH patients vs. 8/21 (38%) TCH patients required a dose delay, 1/16 (6%) FEC-DH patients vs. 5/21 (24%) TCH patients required early cessation of treatment and 6/16 (38%) FEC-DH patients vs. 13/21 (62%) TCH patients required hospitalisation.

## 4. Discussion

The purpose of this audit was to gain a better understanding of the use of NACT for early breast cancer in Hawke’s Bay by evaluating pCR rates and treatment toxicity. A previous larger study of 221 patients found pCR rates in HER2+/HR- disease of 45%, pCR rates in HER2+/HR+ disease of 33% and pCR rates in TNBC disease of 35% [32], and smaller studies have shown similar pCR outcomes [2]. The pCR rates of this audit (62% in HER2+/HR- disease, 34% in HER2+/HR+ disease and 33% in TNBC) are reassuringly in keeping with the findings from the previous literature.

Both severe toxicity and treatment-limiting toxicity were common across all patients treated with NACT. Severe diarrhoea was particularly frequent in patients treated with TCH-based therapy and occurred in 38% of cases, but it is important to note that two (10%) of these patients also received pertuzumab, which is known to cause diarrhoea [33]. While diarrhoea in TCH therapy is well documented, previous research had found that grade 3 severity diarrhoea occurred in only 11% [34] and 5% [35] of neoadjuvant TCH cases, respectively. Therefore, the results from this audit appear high, even with exclusion of patients who received pertuzumab, although it is noted that the number of patients in this audit is relatively small.

In patients who received FEC-DH-based therapy, the most common severe toxicity was neutropaenia (afebrile) which was present in 50% of patients. Research from the literature has shown that neoadjuvant FEC-DH is typically administered with additional pertuzumab [36], and pertuzumab has been found to increase risk of neutropaenia [37]; therefore, there is a lack of directly comparable evidence to assess this result. Despite afebrile neutropaenia occurring in half of FEC-DH-based patients, only one patient required treatment limitation, so this had little impact on clinical management, and all patients were administered prophylactic PEG-filgrastim, with full recovery in their neutrophil counts.

In HER2+ patients, although the proportion of patients who experienced treatment-limiting toxicity was similar for each group (81% for FEC-DH and 76% for TCH), TCH-based therapy led to more dose delays (38.1% vs. 6.3%), earlier treatment cessation (23.8% vs. 6.3%) and more hospitalisations (61.9% vs. 37.5%) than FEC-DH-based therapy, with similar rates of dose reductions in both groups (61.9% in TCH vs. 56.3% in FEC-DH). One potential explanation for this may be that the TCH regimen includes six cycles of docetaxel, whereas FEC-DH only includes three cycles. As discussed previously, reduced chemotherapy dosing intensity from dose reductions and early cessation can negatively impact outcomes, so these findings may have clinical relevance. The pCR rates for TCH-based and FEC-DH-based groups were 33.3% and 43.8%, respectively, and the increased rates of treatment limitations in the TCH-based group may have played a role in this reduction in pCR rate, but the small sample number in this study prevents overinterpretation of this hypothesis.

However, it is also important to consider that FEC-DH contains the anthracycline agent epirubicin, a class of drug that can be associated with significant long-term toxicity. This includes increased risk of acute myeloid leukaemia and myelodysplastic syndrome [28,29], as well as higher incidences of congestive cardiac failure, which can develop many years after treatment [26,38]. This cardiotoxicity is of particular significance given that all HER2+ disease patients will also require a HER2+ targeted therapy such as trastuzumab, which is also associated with impaired heart function [27]. Therefore, it is important to consider all toxicity, both immediate and distant, when determining the best NACT regimen for each patient. Due to the nature of this study, long-term side effects could not be assessed.

There are several limitations to this audit. Due to the relatively small patient numbers overall, it is difficult to draw conclusions about the effectiveness of one regimen over another. This audit only includes patients treated since 2019, and therefore the timeframe is insufficient to enable EFS and OS to be assessed, resulting in pCR being relied upon as a surrogate for long-term outcomes. NACT regimens with a common treatment base (FEC-D, FEC-DH, TCH) were combined with the aim of enabling meaningful comparison between groups; however, this makes it challenging to interpret the toxicity effects of the additional agents such as pertuzumab added to TCH/FEC-DH. Pertuzumab is known to cause diarrhoea [33], and 2/8 TCH-based patients who developed severe diarrhoea also received pertuzumab, which may have been the true cause of their diarrhoea.

The assessment of toxicity relied upon the clinical documentation in oncology clinic letters. There was variability in the level of detail recorded depending on the clinician, which lead to a degree of subjectivity in the reader’s interpretation and raised potential for misclassification bias. Attempts were taken to mitigate this by having a single reader for all clinic letters to improve consistency and using the CTCAE scoring tool wherever possible. Recording treatment-limiting toxicity provided a more reliable measure of toxicity significance, as toxicity that was sufficiently concerning for an oncologist to alter the treatment regimen was usually clearly documented and therefore more objective. All NACT regimens have additional medications such as antiemetics and PEG-Filgrastim which can also cause side effects, so attributing toxicity specifically to NACT could be challenging in some cases. The patients in this audit were not randomised to different NACT regimens, as the oncologists selected the optimum regimen for each individual based on factors such as patient fitness, co-morbidities, etc., and therefore a degree of selection bias is inevitable. Despite documentation being incomplete, particularly in the FEC-DH group, there does not appear to be any significant differences in the patient ECOG performance status between the main treatment groups.

Following this audit, one key area for potential improvement in treatment is in the management of diarrhoea in patients receiving TCH +/− pertuzumab. There is evidence that incorporating regular loperamide into the supportive medication regimen for TCH plus lapatinib (a kinase inhibitor) can reduce rates of diarrhoeal events [34], and introducing a similar approach for TCH treatment in HB may help to reduce incidences of severe diarrhoea. From further analysis into patients who were hospitalised secondary to diarrhoea, it was noted that two patients had uncontrolled diarrhoea symptoms for one week prior to presenting to hospital. This raises the possibility that introducing additional telephone follow-up appointments to ensure that these patients have optimal loperamide education and supplies may help to reduce hospitalisation.

## 5. Conclusions

This audit has shown that response rate for NACT in early breast cancer in HB is consistent with the results from wider research. Toxicities from NACT were common and frequently led to treatment alterations and hospitalisation. With the caveat of small patient numbers, FEC-DH-based therapy is observed to be associated with fewer dosing delays, early treatment cessations and hospitalisations compared with TCH-based therapy but comes with potential additional risks of long-term toxicities beyond the scope of this audit. Management of diarrhoea in TCH-based therapy is an area for potential improvement, and having additional telephone follow-up to optimise supportive loperamide treatment may improve future patient care.

## Figures and Tables

**Table 1:** pCR rates by molecular subtype.

Molecular Subtype	TNBC	HER2+ (Overall)	HER2+/HR-	HER2+/HR+	HR+/HER2−
pCR rate	8/24 (33%)	19/45 (42%)	8/13 (62%)	11/32 (34%)	0/3 (0%)

**Table 2:** NACT pCR rate per molecular subtype, regimen and treatment base.

Molecular Subtype	pCR Rate *	Regimen ^	pCR Rate *	Treatment Base	pCR Rate *
TNBC	8/24 (33.3%)	AC + P	0/1	Non-FEC-D	0/2 (0%)
		C/P/Pem + AC	0/1		
		FEC-D	1/6	FEC-D	8/25 (32%)
		FEC-DC	5/13		
		FEC-DC + Pem	1/2		
		FEC-DC + Azet	1/1		
HR+/HER2−	0/3 (0%)	FEC-D	0/3		
HER2+	19/45 (42.2%)	AC + P/H	0/1	P/H	5/8 (62.5%)
		P/H	4/6		
		P/H + Pert	1/1		
		FEC-DH	5/14	FEC-DH	7/16 (43.8%)
		FEC-DH + Pert	2/2		
		TCH	5/18	TCH	7/21 (33.3%)
		TCH + Pert	2/3		

* pCR rate = Number of pCR patients/number of total patients. ^ Regimens: AC + P = 4 × 2-weekly doxorubicin and cyclophosphamide followed by 12 × weekly paclitaxel; C/P/Pem + AC = 12 × weekly carboplatin and paclitaxel with 3-weekly pembrolizumab followed by 4 × 3-weekly doxorubicin and cyclophosphamide; FEC-D = 3 × 3-weekly 5-fluorouracil, epirubicin and cyclophosphamide followed by 3 × 3-weekly docetaxel; FEC-DC = FEC-D with carboplatin in addition for last three cycles; FEC-DC + Pem = FEC-DC with 3-weekly pembrolizumab; FEC-DC + Aze = FEC-DC with 3-weekly atezolizumab; AC + P/H = 4 × 3-weekly doxorubicin and cyclophosphamide followed by 12 × weekly paclitaxel with 3-weekly trastuzumab; P/H = 12 × weekly paclitaxel with 3-weekly trastuzumab; P/H + Pert = P/H with additional 3-weekly pertuzumab; FEC-DH = FEC-D with 3-weekly trastuzumab in addition for last three cycles; FEC-DH + Pert = FEC-DH with 3-weekly pertuzumab in addition for last three cycles; TCH = 6 × 3-weekly docetaxel, carboplatin and trastuzumab; TCH + Pert = TCH with additional 3-weekly pertuzumab.

**Table 3:** ECOG performance status per NACT treatment base.

Treatment Base	FEC-D	FEC-DH	TCH
Total patients	25	16	21
ECOG Performance status			
0	17 (68%)	8 (50%)	12 (57%)
1	6 (24%)	1 (6%)	8 (38%)
2	0 (0%)	1 (6%)	1 (5%)
Not documented	2 (8%)	6 (38%)	0 (0%)

**Table 4:** Severe toxicity per NACT treatment base.

Treatment Base	FEC-D	FEC-DH	TCH
Total patients	25	16	21
Patients with severe toxicity	16	12	14
Severe toxicity percentage	64% (16/25)	75% (12/16)	67% (14/21)
Toxicity		Number of patients (%)	
Diarrhoea	4 (16%)	1 (6%)	8 (38%)
Neutropaenia (afebrile)	3 (12%)	8 (50%)	1 (5%)
Anaemia	4 (16%)	1 (6%)	4 (19%)
Febrile neutropaenia	4 (16%)	1 (6%)	2 (10%)
Lung infection	1 (4%)	2 (13%)	2 (10%)
Mucositis	2 (8%)	0 (0%)	3 (14%)
Fever	2 (8%)	3 (19%)	0 (0%)
Vomiting	1 (4%)	1 (6%)	1 (5%)
Anorexia	1 (4%)	1 (6%)	1 (5%)
Aches *	0 (0%)	3 (19%)	0 (0%)
Colitis	0 (0%)	0 (0%)	2 (10%)
Device-related infection	2 (8%)	0 (0%)	0 (0%)
Hyperglycaemia	1 (4%)	0 (0%)	1 (5%)
Dyspnoea	1 (4%)	0 (0%)	1 (5%)

* Aches include arthralgia, myalgia and bone pains.

**Table 5:** Treatment-limiting toxicity per NACT treatment base.

Treatment Base	FEC-D	FEC-DH	TCH
Total patients	25	16	21
Patients with treatment-limiting toxicity	12	13	16
Treatment-limiting toxicity percentage	48% (12/25)	81% (13/16)	76% (16/21)
Toxicity		Number of patients (%)	
Diarrhoea	4 (16%)	3 (19%)	5 (24%)
Fatigue	5 (20%)	2 (13%)	4 (19%)
Neuropathy	1 (4%)	2 (13%)	4 (19%)
Nausea	2 (8%)	1 (6%)	3 (14%)
Anorexia	2 (8%)	1 (6%)	2 (10%)
Febrile neutropaenia	4 (16%)	0 (0%)	2 (10%)
Cardiotoxicity (decline in LVEF)	0 (0%)	2 (13%)	2 (10%)
Lung infection	1 (4%)	0 (0%)	1 (5%)
Anaemia	2 (8%)	0 (0%)	1 (5%)
Aches *	0 (0%)	4 (25%)	0 (0%)
Vomiting	1 (4%)	1 (6%)	1 (5%)
Neutropaenia (afebrile)	2 (8%)	1 (6%)	0 (0%)
Mucositis	1 (4%)	0 (0%)	1 (5%)
Colitis	0 (0%)	0 (0%)	2 (10%)
DKA	1 (4%)	0 (0%)	1 (5%)

* Aches include arthralgia, myalgia and bone pains.

## Data Availability

No new data were created or analysed in this study. Data sharing is not applicable to this article.
